# Association between ambient air pollutants and preterm birth in Ningbo, China: a time-series study

**DOI:** 10.1186/s12887-018-1282-9

**Published:** 2018-09-20

**Authors:** Wen-Yuan Liu, Zhe-Bin Yu, Hai-Yan Qiu, Jian-Bing Wang, Xue-Yu Chen, Kun Chen

**Affiliations:** 1 Key laboratory of maternal-fetal medicine, Ningbo Women and Children’s Hospital, Ningbo, 315012 China; 2 Department of Epidemiology and Biostatistics, School of Public Health, Zhejiang University, Hangzhou, 310058 China; 3 Research Center for Air Pollution and Health, Zhejiang University, Hangzhou, 310058 China

**Keywords:** Preterm birth, Air pollution, Time-series analysis, PM_2.5_, PM_10_, SO_2_

## Abstract

**Background:**

Exposure to air pollutants has been related to preterm birth, but little evidence can be available for PM_2.5_, O_3_ and CO in China. This study aimed to investigate the short-term effect of exposure to air pollutants on risk preterm birth during 2014–2016 in Ningbo, China.

**Methods:**

We conducted a time-series study to evaluate the associations between daily preterm birth and major air pollutants (including PM_2.5_, PM_10_, SO_2_, NO_2_, O_3_ and CO) in Ningbo during 2014–2016. A General Additive Model extend Poisson regression was used to evaluate the relationship between preterm birth and air pollution with adjustment for time-trend, meteorological factors and day of the week (DOW). We also conducted a subgroup analysis by season and age.

**Results:**

In this study, a total of 37,389 birth occurred between 2014 and 2016 from the Electronic Medical Records System of Ningbo Women and Children’s Hospital, of which 5428 were verified as preterm birth. The single pollutant model suggested that lag effect of PM_2.5_, PM_10_, NO_2_ reached a peak at day 3 before delivery and day 6 for SO_2_, and no relationships were observed for O_3_ and preterm birth. Excess risks (95% confidence intervals) for an increase of IQR of air pollutant concentrations were 4.84 (95% CI: 1.77, 8.00) for PM_2.5_, 3.56 (95% CI: 0.07, 7.17) for PM_10_, 3.65 (95% CI: 0.86, 6.51) for SO_2_, 6.49 (95% CI: 1.86, 11.34) for NO_2_, − 0.90 (95% CI: -4.76, 3.11) for O_3_, and 3.36 (95% CI: 0.50, 6.30) for CO. Sensitivity analyses by exclusion of maternal age < 18 or > 35 years did not materially alter our results.

**Conclusions:**

This study indicates that short-term exposure to air pollutants (including PM_2.5_, PM_10_, SO_2_, NO_2_) are positively associated with risk of preterm birth in Ningbo, China.

**Electronic supplementary material:**

The online version of this article (10.1186/s12887-018-1282-9) contains supplementary material, which is available to authorized users.

## Background

Preterm birth, defined as less than 37 weeks of gestations, is the second largest direct cause of child deaths among children less than 5 years [1]. There are 15 million premature birth annually worldwide and China contributed 1.1 million (rank 2nd worldwide) according to international survey data [2]. Preterm birth account for 75% of perinatal mortality and more than half the long-term morbidity [3]. Moreover, the survived preterm babies are at increased risk of neuro-developmental impairments, respiratory and gastrointestinal complications [3]. The etiology of preterm birth remains unclear yet many risk factors have been explored.

There is increasing evidence that exposure to ambient air pollutants is associated with preterm birth [4–9]. A systematic review has reported positive associations between air pollutants and risk of adverse birth outcomes including preterm birth [5]. And a recent meta-analysis of 23 studies has also showed that a significantly increased risk of preterm birth with interquartile range increase in particulate matter exposure during pregnancy [10]. It should be noted that findings of exposure to air pollution and preterm birth from Western countries may not be applicable to the Chinese populations due to higher air pollution levels, genetic and physiological differences. However, a recent systematic review, included all studies in China, showed the effect of air pollution on preterm birth was inconsistent [11].

In this study, we used birth data during 2014–2016 in Ningbo, Zhejiang Province, China, and conducted a time-series study to investigate the association between exposure to ambient air pollutants and risk of preterm birth.

## Methods

### Study population

This study was conducted in Ningbo, which located in the southeast of China and composed of six districts and has a metropolitan area population of 7.8 million. We obtained anonymous births information from the Electronic Medical Records System (EMRS) in Ningbo Women and Children’s Hospital (the largest women’s hospital in Ningbo) from 2014 January 1st to 2016 December 31st. A total of 40,968 birth records were included in the EMRS. Duplicated records (*n* = 2305), non-live birth records (*n* = 230), twin pregnancy and multiple pregnancies (*n* = 1274) and birth records with extreme gestational age (< 20 weeks) (*n* = 160) were excluded from this study. Finally, a total of 37,389 eligible births were included in our study.

### Preterm birth

Preterm birth was defined as a singleton live-birth delivery before 37 completed weeks of gestation(< 259 days) [1]. Gestational age was calculated based on the date of women’s last menstrual period (LMP). For women who had no LMP date, gestational age was substituted by a clinical estimate. A total of 5428 preterm births were finally included for the current analysis. The number of preterm births was calculated for each day from 2014 January 1st to 2016 December 31st. The study was reviewed and approved by Committee of ethics, Ningbo Women and Children’s Hospital.

### Air pollution and meteorological exposure

Daily meteorological data including mean temperature (degree Celsius) and relative humidity(percent) were collected from the Ningbo Meteorological Bureau. Daily values for temperature and relative humidity were calculated by averaging 24 hourly monitoring data.

Daily mean concentrations of air pollutants, including particulate matter (aerodynamic diameter less than or equal to 2.5 μm (PM_2.5_) and 10 μm (PM_10_)), sulfur dioxide (SO_2_), nitrogen dioxide (NO_2_), Ozone (O_3_) and carbon monoxide (CO) during 2014 to 2016, were collected from the Environmental Monitoring Center of Ningbo City (http://www.nbemc.net/aqi/home/index.aspx). The daily concentrations of each pollutant were averaged from the available monitored results of eight stations which were monitored by the China National Quality Control. The eight stations were “Shi Jian Ce Zhong Xin”, “Tai Gu Xiao Xue”, “San Jiang Zhong Xue”, “Wan Li Xue Yuan”, “Huan Bao Da Lou”, “Long Sai Yi Yuan”, “Qian HuShui Chang” and “Wan Li Guo Ji”. The distribution of these 8 monitor stations in Ningbo was shown in Additional file 1: Figure S1. Air pollutants were measured in the unit of micrograms per cubic meter(μg/m^3^) except milligrams per cubic meter (mg/m^3^) for CO.

### Statistical analysis

Distribution of daily number of preterm births follows the Poisson distribution due to its small probabilities. Thus, we used a Generalized Additive Model (GAM) extended Poisson regression [12] to explore the potential effect of air pollution on premature birth. This method has been widely used in air pollution time-series studies [13–22] because of its non-parametric flexibility.

We firstly built a basic model based on the daily number of preterm births without air pollution variables. To control for non-linear trend between preterm birth and time or weather conditions, we added time-dependent variables including calendar time, temperature and relative humidity via natural spline functions. Degree of freedom (df) for natural spline functions were adopted by generalized cross-validation (GCV) scores [12]. Day of the week was also included as a dummy variable in the basic models. Then, each air pollutant was added into a single-pollutant model separately. The number of gestations at risk of preterm birth was used as an offset. In brief, we fitted the following model to evaluate the effect of air pollutants on preterm birth:

$$ \mathrm{Log}\left[\mathrm{E}\left({\mathrm{Y}}_{\mathrm{t}}\right)\right]=\upalpha +{\upbeta \mathrm{Z}}_{\mathrm{t}}+\mathrm{S}\kern0.5em \left(\mathrm{time},\kern0.5em \mathrm{df}\right)+\mathrm{S}\kern0.5em \left(\mathrm{temperature},\kern0.5em \mathrm{df}\right)+\mathrm{S}\kern0.5em \left(\mathrm{relative}\kern0.5em \mathrm{humidity},\kern0.5em \mathrm{df}\right)+{\mathrm{DOW}}_{\mathrm{t}}\left(\mathrm{day}\kern0.5em \mathrm{of}\kern0.5em \mathrm{the}\kern0.5em \mathrm{week}\right)+{\mathrm{Offset}}_{\mathrm{t}} $$

In this formula, *t* represents the day of the observation; Y_t_ represents daily number of preterm births, E(Y_t_) stands for the expected values for the number of premature births on day *t.* α is residual, β is the regression coefficient, and Z_t_ is the average concentration of air pollutants on the observed day or over several days. S (time, df) is the calendar time smoothing spline function, S (temperature, df) is the daily temperature smoothing spline function, S (relative humidity, df) is the daily relative humidity smoothing spline function, and DOW_*t*_ is a dummy variable with Monday as a reference. The corresponding degree of freedom for time, temperature and relative humidity in the spline function were 7, 7 and4 in the final model.

We investigated the acute effect on the risk of preterm birth by adding the concentration of each pollutant into the model for a 1-day exposure window with lag-time from 1 to 6 days before birth. Cumulative effect was also calculated by including the lag moving average (Avg1-Avg6) into the model. Relative risks (RRs) and 95% confident intervals (CIs) were calculated by the regression coefficient β of air pollutants. And we reported excess risks (ERs) and 95% CIs that represented a percent increase in daily preterm birth risk per IQR increase in air pollutant concentrations. ER was calculated as follows: ER = (RR ‐ 1) × 100%. We also examine the exposure-response curve by using a natural spline function for certain pollutants in the GAM model. Goodness of fit of the model was assessed by using Akaike Information Criterion (AIC). The best df for each air pollutant was indicated by the lowest AIC value in the GAM model.

Sensitivity analysis by exclusion of maternal age < 18 or > 35 years in preterm birth records was conducted to evaluate the robustness of our results, because women aged < 18 or > 35 years had a higher possibility to develop a preterm birth [23]. And we further divided the study period into cold period (November to April) and warm period (May to October). Models were fitted separately in two periods to check if any difference in the effect of air pollutants on preterm birth during warm and cold periods. 95% confidence interval for the difference in effect estimates between two strata (a potential effect modifier) was calculated as follows:

$$ \left(\mathrm{Q}1\hbox{-} \mathrm{Q}2\pm 1.96\sqrt{\mathrm{SE}1+\mathrm{SE}2}\right) $$

Where Q1 and Q2 are the adjusted estimates from two strata (e.g. cold and warm period), and SE1, SE2 are the corresponding standard errors [24].

Continuous variables with normal distribution were presented as mean ± standard deviation (SD), and non-normal variables were reported as median ± interquartile range (IQR). Spearman’s correlation coefficient was used for the correlations between ambient air pollutants and meteorological factors. *P* < 0.05 was considered statistically significant. All statistical analyses were conducted by using R 3.3.1.

## Results

### Descriptive results of exposure and outcomes

The descriptive results of air pollution and meteorological data are shown in Table 1. The mean daily concentrations of PM_2.5_, PM_10_, SO_2_, NO_2_, O_3_ and CO during 2014 to 2016 were 43.73 μg/m^3^, 69.69 μg/m^3^, 16.56 μg/m^3^, 40.50 μg/m^3^, 64.33 μg/m^3^, 1.06 mg/m^3^, respectively. Concentrations of air pollutants were higher in the cold period than those in the warm period except for O_3_. Daily mean ambient temperature and relative humidity were 17.4 °C and 76.8%.

**Table 1:** Air pollution and meteorological data in Ningbo, China (2014–2016)

	Mean ± SD			Minimum	P_25_	P_50_	P_75_	IQR	Maximum
	ALL year	Cold Period^a^	Warm Period						
Air pollutants									
SO_2_ (μg/m^3^)	16.56 ± 9.05	18.91 ± 10.25	14.25 ± 6.97	5.90	10.53	13.70	19.09	8.56	74.08
NO_2_ (μg/m^3^)	40.50 ± 16.88	49.85 ± 15.99	31.28 ± 11.94	5.59	28.00	37.38	51.34	23.34	115.00
PM_10_ (μg/m^3^)	69.69 ± 38.37	87.03 ± 41.75	52.60 ± 24.85	10.18	42.90	60.20	85.31	42.41	287.10
PM_2.5_ (μg/m^3^)	43.73 ± 26.26	55.64 ± 29.38	31.99 ± 15.55	4.24	25.50	37.38	54.11	28.62	196.93
O_3_ (μg/m^3^)	64.33 ± 29.71	53.46 ± 25.07	75.05 ± 30.05	8.17	42.99	61.96	83.02	40.03	244.30
CO (mg/m^3^)	1.06 ± 0.35	1.13 ± 0.39	0.99 ± 0.28	0.04	0.88	1.00	1.19	0.31	2.92
Meteorology									
Temperature (°C)	17.42 ± 8.10	10.64 ± 5.17	24.10 ± 3.76	−4.47	10.23	18.69	23.94	13.71	32.25
Relative Humidity (%)	76.8 ± 11.80	74.29 ± 13.38	79.19 ± 9.33	32.96	69.82	77.81	85.47	15.65	97.60

*PM*_*2.5*_: particulate matter less than 2.5 μm in aerodynamic diameter, *PM*_*10*_: particulate matter less than 10 μm in aerodynamic diameter, *SO*_*2*_: sulfur dioxide, *NO*_*2*_: nitrogen dioxide, *O*_*3*_: Ozone, *CO*: carbon monoxide

^a^Cold period was from November to April, and warm period was from May to October

A total of 5428 preterm births were identified among the total valid births of 37,159. Overall prevalence of preterm birth was 14.61%. The number of births in women with the maternal age < 18 or > 35 years was 3452, among which 714 births were diagnosed as preterm birth (20.68%). And the corresponding prevalence of preterm birth during cold and warm periods was 14.63% and 14.58%, respectively.

### Correlation between ambient air pollutants and meteorological factors

Table 2 shows the Spearman’s correlation analysis of air pollution and meteorological measures. PM_2.5_ was positively associated with SO_2_, NO_2_, PM_10_ and CO, but negatively associated with O_3_. The strong correlation was observed for PM_2.5_ and NO_2_ (Spearman’s Rho = 0.74, *P* < 0.01). And two weather variables were negatively related to SO_2_, NO_2_, PM_2.5_, PM_10_ and CO, but positively related to Ozone.

**Table 2:** Correlation between air pollutants and meteorological factors in Ningbo, China

	SO_2_	NO_2_	PM_10_	PM_2.5_	CO	O_3_	Temperature	Relative humidity
SO_2_	1.00							
NO_2_	0.59	1.00						
PM_10_	0.69	0.74	1.00					
PM_2.5_	0.66	0.74	0.95	1.00				
CO	0.22	0.45	0.43	0.47	1.00			
O_3_	−0.13	−0.46	−0.13	−0.17	− 0.29	1.00		
Temperature	−0.39	− 0.62	− 0.52	−0.50	− 0.20	0.37	1.00	
Relative humidity	−0.39	−0.04	− 0.36	−0.24	0.08	−0.33	0.21	1.00

*PM*_*2.5*_: particulate matter less than 2.5 μm in aerodynamic diameter, *PM*_*10*_: particulate matter less than 10 μm in aerodynamic diameter, *SO*_*2*_: sulfur dioxide, *NO*_*2*_: nitrogen dioxide, *O*_*3*_: Ozone, *CO*: carbon monoxide

All correlations were statistically significant (*P* < 0.01)

### Short-term effects for preterm birth

**Fig. 1:**
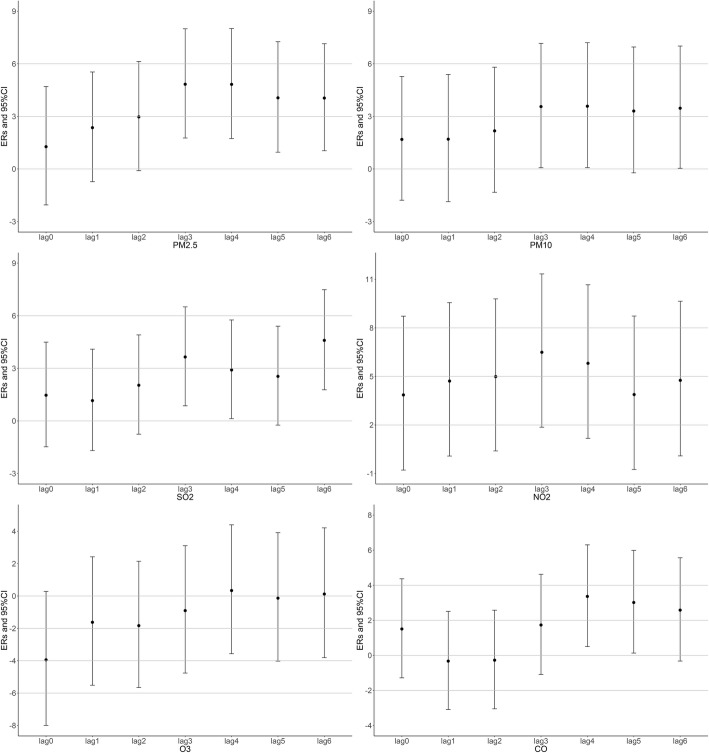
Excess Risks (ERs) and 95% confidence intervals (95% CIs) of daily preterm birth risk per IQR increment in pollutant concentrations at different lag days

**Fig. 2:**
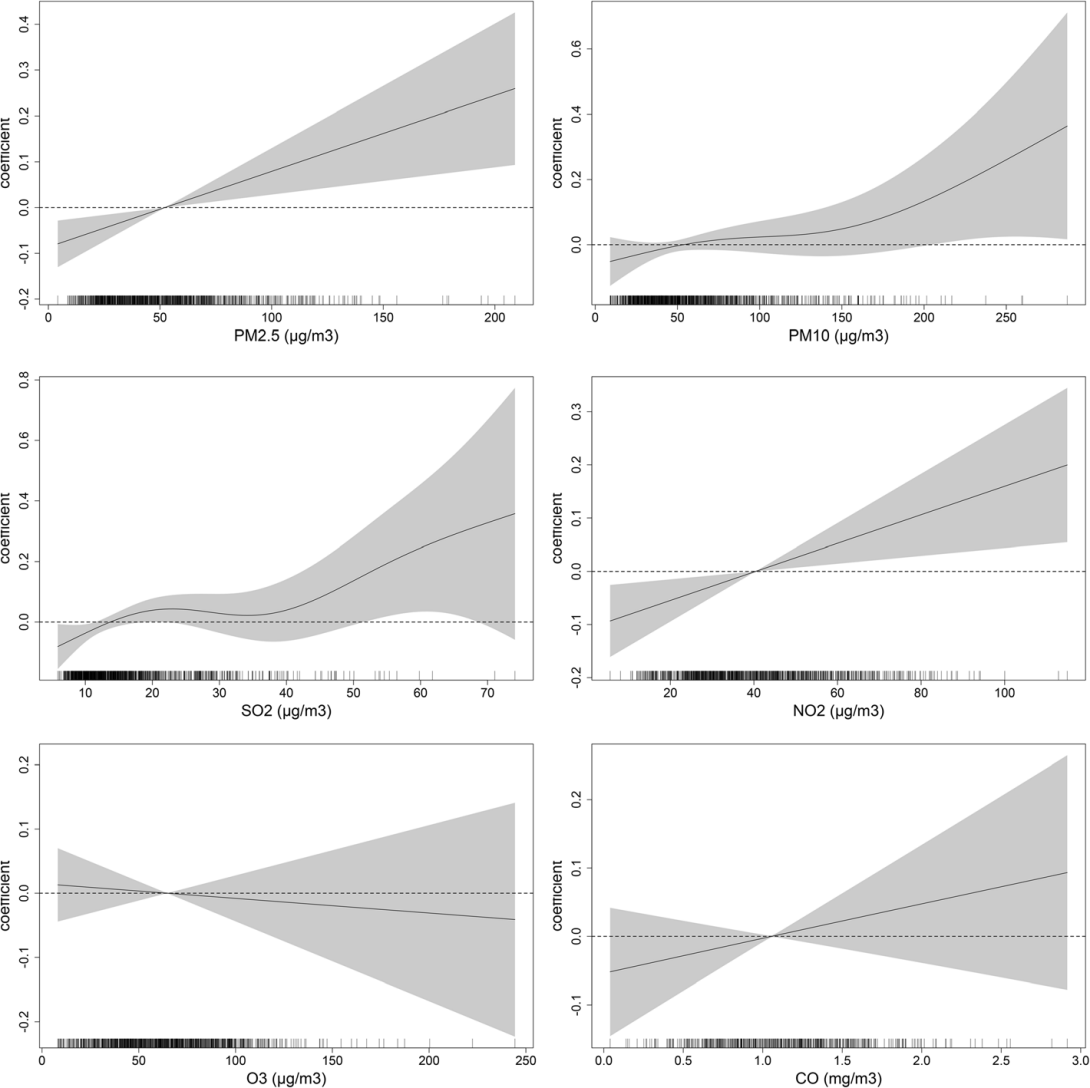
Coefficients and 95% confidence intervals (95% CIs) of daily preterm birth risk at different pollutant concentrations using natural spline functions

Fig 1 shows the association between air pollutants and daily preterm births at lag0–6 days. The largest ERs were observed at Lag3 for PM_2.5_, PM_10_ and NO_2_, Lag6 for SO_2_ and Lag 4 for CO. No significant associations were observed for Ozone and preterm births. The associations between cumulative concentrations and preterm births at different lag days (Avg1-Avg6) are shown in the Additional file 2: Table S1. Figure 2 shows the dose-response curve between certain air pollutants and risk of preterm births by using a natural spline function for air pollutants in GAM models. Nonlinear association was observed for PM_10_, SO_2_ and preterm births.

Table 3 and Additional file 3: Table S2 show the excess risks and 95% CIs for short-term exposure to air pollutants and daily preterm birth stratified by maternal age and season. The associations between PM_2.5_, PM_10_, SO_2_, NO_2_ and preterm birth tended to be attenuated after we restricted the analysis in women with the maternal age of 18–35 years, but the associations still remained significant. In season-specific analyses, the adverse effect of PM_2.5_, SO_2_ and NO_2_ on preterm birth were stronger in cold period and attenuated in warm period as compared with the whole year. Similar results were observed for the effect of four air pollutants (PM_2.5_, PM_10_, SO_2_ and NO_2_) in cold and warm periods when maternal age was restricted from 18 to 35 years. No significant associations were observed for Ozone. No significant interaction effect was observed for season and maternal age on the association of short-term exposure to air pollution and preterm birth (Additional file 4: Table S3).

**Table 3:** Excess risks (ERs) and 95% confidence intervals of preterm birth per IQR increment in air pollutant concentrations in warm and cold periods in Ningbo, China

	All births			Gestational women age between 18 and 35			Gestational women under 18 or above 35		
	Annual	Cold period	Warm period	Annual	Cold period	Warm period	Annual	Cold period	Warm period
PM_2.5_^b^	*4.84 (1.77,8.00)* ^c^	*4.92 (1.47,8.50)* ^c^	4.06 (− 2.55,11.12)	*4.53 (1.09,8.09)* ^c^	*4.65 (0.71,8.74)* ^c^	3.17 (− 4.23,11.15)	*9.40 (0.74,18.80)* ^c^	*12.24 (2.40,23.04)* ^c^	− 1.93 (− 18.95,18.67)
PM_10_^b^	*3.56 (0.07,7.17)* ^c^	*5.11 (1.04,9.35)* ^c^	1.87 (−5.26,9.53)	*4.88 (0.88,9.03)* ^c^	*6.74 (2.12,11.58)* ^c^	2.64 (− 5.42,11.39)	*13.22 (2.55,25.00)* ^c^	*16.73 (4.50,30.40)* ^c^	0.65 (−19.38,25.66)
SO_2_^b^	*3.65 (0.86,6.51)* ^c^	*4.93 (1.64,8.33)* ^c^	1.44 (− 3.70,6.85)	*6.56 (3.39,9.82)* ^c^	*7.04 (3.29,10.92)* ^c^	5.53 (−0.32,11.71)	*8.46 (0.21,17.40)* ^c^	*10.95 (1.37,21.45)* ^c^	2.79 (− 12.58,20.87)
NO_2_^b^	*6.49 (1.86,11.34)* ^c^	*9.25(3.45,15.38)* ^c^	3.12(−5.08,12.02)	*6.66(1.42,12.17)* ^c^	*10.32(3.69,17.37)* ^c^	2.7(−6.26,12.51)	*14.20 (1.18,28.90)* ^c^	7.40(−7.79,25.09)	−2.94(− 23.32,22.87)
O_3_^b^	− 0.90 (− 4.76,3.11)	−6.35 (− 12.78,0.55)	3.23 (− 1.81,8.53)	− 2.76 (− 7.1,1.79)	− 3.86 (− 10.69,3.49)	1.55 (− 3.91,7.32)	−4.11 (− 14.14,7.10)	−8.96 (− 24.85,10.28)	−1.23 (− 14.53,14.15)
CO^b^	*3.36 (0.50,6.30)* ^c^	*5.16 (1.33,9.13)* ^c^	1.89 (−2.71,6.71)	0.37 (−2.84,3.70)	2.29 (− 1.16,5.87)	0.40 (− 4.60,5.66)	*7.82 (0.85,15.28)* ^c^	*10.44 (1.71,19.92)* ^c^	4.14 (−7.37,17.08)

*PM*_*2.5*_: particulate matter less than 2.5 μm in aerodynamic diameter, *PM*_*10*_: particulate matter less than 10 μm in aerodynamic diameter, *SO*_*2*_: sulfur dioxide, *NO*_*2*_: nitrogen dioxide, *O*_*3*_: Ozone, *CO*: carbon monoxide

^a^ERs were calculated per IQR increment for each air pollutant

^b^Lag day (lag 3 for PM_2.5_, PM_10_, NO_2_, SO_2_, O_3_, lag4 for CO) were used

^c^*P*<0.05

Table 4 provides ERs and 95% CIs from two-pollutant models. The effect of air pollutants on daily preterm birth became nonsignificant after controlling for other air pollutants in the two-pollutant models.

**Table 4:** Excess risks (ERs) and 95% confidence intervals (CIs) of daily preterm birth in two-pollutant models

	Two-pollutant models^a^	ERs and 95% CIs
PM_2.5_	–	4.84 (1.77, 8.00)
	NO_2_	3.44 (−0.47, 7.50)
	SO_2_	3.70 (−0.02, 7.52)
PM_10_	–	3.56 (0.07, 7.17)
	NO_2_	0.42 (−4.13,5.20)
	SO_2_	1.15 (−3.21, 5.69)
SO_2_	–	3.65 (0.86, 6.51)
	PM_2.5_	1.69 (−1.55, 5.08)
	PM_10_	3.09 (−0.41, 6.72)
	NO_2_	2.18 (−1.07,5.52)
NO_2_	–	6.49 (1.86, 11.34)
	PM_2.5_	3.09 (−3.61, 9.11)
	PM_10_	6.11 (−0.03,12.62)
	SO_2_	4.55 (−0.80, 10.18)

*PM*_*2.5*_: particulate matter less than 2.5 μm in aerodynamic diameter, *PM*_*10*_: particulate matter less than 10 μm in aerodynamic diameter, *SO*_*2*_: sulfur dioxide, *NO*_*2*_: nitrogen dioxide

^a^Lag day 3 for PM_2.5_, PM_10_, NO_2_, SO_2_ were used

## Discussion

In this study, we performed an ecological time-series study to examine the short-term effect of air pollutants on preterm birth during 2014–2016 in Ningbo. We found that PM_2.5_, PM_10_, SO_2_ and NO_2_ were significantly associated with increased risk of preterm birth during 1-week preceding delivery. Single pollutant analysis using General Additive Model indicated that the effect of PM_2.5_, PM_10_, and NO_2_ reached a peak value at lag day 3 and SO_2_ at lag day 6. The corresponding ERs for an increased concentration of IQR were 4.84 (95% CI: 1.77, 8.00) for PM_2.5_, 3.56 (95% CI: 0.07, 7.17) for PM_10_, 3.65 (95% CI: 0.86, 6.51) for SO_2_, and 6.49 (95% CI: 1.86, 11.34) for NO_2_, respectively.

The observed effect of particulate matter (PM_2.5_, PM_10_) were consistent with several previous studies [13, 25–29]. A ten- year time-series study conducted in Rome [30] has detected a significant effect of PM_10_ on preterm-birth risk. An updated meta-analysis 10 of 23 studies has showed an increased risk with an IQR increase in PM_10_ exposure during pregnancy (pooled OR = 1.03, 95% CI:1.01–1.05). Limited studies in China can be available to evaluate the effect of PM exposure on preterm birth. A birth cohort conducted in Lanzhou, China between 2010 and 2012 [26] also found that exposure to high levels of ambient PM_10_ could increase the risk of preterm birth, and another prospective birth cohort in China confirmed the adverse effect on preterm birth risk of PM_2.5_ exposure [23]. Our study also indicated significant associations for PM_2.5_, PM_10_ exposure and preterm birth, but the RRs were relatively lower. The discrepancies could be explained by the different design, population and particulate matter level.

Our study found significant associations between maternal exposure to SO_2_, NO_2_ and preterm birth 1 week proceeding delivery. SO_2_ was consistently associated with preterm birth according to a systematic review of 25 studies conducted in China [11]. Previous time-series studies conducted in China and Atlanta, USA also observed that increased NO_2_ concentration was associated with preterm birth risk [13, 27]. The effects of O_3_ and CO were less well-studied because the monitoring network of these air pollutants by Chinese government started from 2013. In our study, we found no significant effect of CO and O_3_ on preterm birth, even after stratified by maternal age and season. However, a previous study reported an increase of 5% in risk of preterm birth per 100 μg/m^3^ increase in CO concentrations in the second trimester of pregnancy [23, 31]. Further studies are needed to confirm the effect of carbon monoxide and the critical windows of exposure to these air pollutants.

In our study, the effect of air pollutants on preterm birth risk tended to be stronger in cold period than that in warm period, although this difference was not statistically significant. Previous studies have also showed that the effect of air pollutants on preterm birth varied in different seasons [32]. This seasonal discrepancy may be explained by a higher level of air pollutants in cold period as Table 1 shows. Furthermore, residents may reduce time to go outdoors due to high temperature and frequent rain during warm seasons [17] thus the chance of exposure to ambient air pollution is relatively lower as compared with cold seasons.

The association between short-term exposure to certain air pollutants and risk of preterm birth may suggest that air pollution can motivate the biologic mechanism of labor and thus leading to preterm birth. Potential mechanisms for this association could be explained by inflammation, endocrine disruption, hemodynamic responses, oxidative stress and endothelial dysfunction [33]. When air pollutants are inhaled into the body, oxidative stress and intrauterine inflammation may induce preeclampsia [34] and preterm premature rupture of membranes [35], which could contribute a significant part of the causes of preterm birth.

Our study had several important strengths. Firstly, we used time-series Generalized Additive Model extended Poisson regression to adjust for the confounding effects of long-time trends, meteorological factors and season. In addition, our study provided evidence for the effect of previously less well-studied air pollutants (O_3_ and CO). Our study also had several limitations. Average data from fixed monitoring locations were used to represent air pollution exposure, which could affect our results. And ecological study design could underestimate the effect of air pollution when monitoring data was used to represent individual exposure level [36]. It should also be noted that our analyses were not adjusted for infant gender, maternal smoking status and education level due to lack of these individual risk factors. Future studies with individual risk factors especially time varying factors (such as maternal smoking exposure) are needed to confirm our findings. Besides, early obstetric ultrasound was used to estimate the gestational age instead of LMP for a small portion of women who forgot their last menstrual period. There are also other hospitals can be selected in the region, but medical records in other hospitals cannot be available in the current study. We believe that these issues would not affect our results. Finally, we cannot identify the independent effect of each pollutant due to high correlations between pollutants.

## Conclusions

In summary, this study examined the association between concentrations of air pollutants (PM_2.5_, PM_10_, SO_2_, NO_2_, O_3_ and CO) and risk of preterm birth in Ningbo. Our results suggested that short-term exposure to four pollutants (PM_2.5_, PM_10_, SO_2_ and NO_2_) were associated with preterm birth risk in Ningbo. These findings might have important implications in preventing preterm birth while further studies are still needed.

## Additional files

Additional file 1: **Figure S1.** Location of air quality monitor stations in Ningbo city. (PDF 13330 kb)

Additional file 2: **Table S1.** Association between cumulative air pollution concentrations and risk of preterm birth. (DOCX 18 kb)

Additional file 3: **Table S2.** Excess risks (ERs) and 95% confidence intervals of preterm birth per IQR increment in air pollutant concentrations stratified by season and maternal age in Ningbo, China. (DOCX 36 kb)

Additional file 4: **Table S3.** Difference of estimates and 95% confidence intervals (95% CIs) of air pollutants on risk of preterm birth between subgroups. (DOCX 20 kb)

## References

[1] Goldenberg RL, Culhane JF, Iams JD, Romero R (2008). Epidemiology and causes of preterm birth. LANCET.

[2] Blencowe H, Cousens S, Oestergaard MZ, Chou D, Moller AB, Narwal R, Adler A, Vera GC, Rohde S, Say L (2012). National, regional, and worldwide estimates of preterm birth rates in the year 2010 with time trends since 1990 for selected countries: a systematic analysis and implications. LANCET.

[3] Muglia LJ, Katz M (2010). The enigma of spontaneous preterm birth. N Engl J Med.

[4] Polichetti G, Capone D, Grigoropoulos K, Tarantino G, Nunziata A, Gentile A (2013). Effects of ambient air pollution on birth outcomes: an overview. Crit Rev Environ Sci Technol.

[5] Stieb DM, Chen L, Eshoul M, Judek S (2012). Ambient air pollution, birth weight and preterm birth: a systematic review and meta-analysis. Environ Res.

[6] Shah PS, Balkhair T, KSGD P (2011). Air pollution and birth outcomes: a systematic review. Environ Int.

[7] Sun X, Luo X, Zhao C, Chung NR, Lim CE, Zhang B, Liu T (2015). The association between fine particulate matter exposure during pregnancy and preterm birth: a meta-analysis. BMC Pregnancy Childbirth.

[8] Liu C, Sun J, Liu Y, Liang H, Wang M, Wang C, Shi T. Different exposure levels of fine particulate matter and preterm birth: a meta-analysis based on cohort studies. Environ Sci Pollut Res Int. 2017. 10.1007/s11356-017-9363-0 28616740

[9] Khader Y, Abdelrahman M, Abdo N, Awad S, Al-Sharif M, Elbetieha A, Malkawi M (2016). Exposure to Air Pollution and Pregnancy Outcomes in the East Mediterranean Region: a Systematic Review. Int J Pediatrics-Mashhad.

[10] Li X, Huang S, Jiao A, Yang X, Yun J, Wang Y, Xue X, Chu Y, Liu F, Liu Y (2017). Association between ambient fine particulate matter and preterm birth or term low birth weight: an updated systematic review and meta-analysis. Environ Pollut.

[11] Jacobs M, Zhang G, Chen S, Mullins B, Bell M, Jin L, Guo Y, Huxley R, Pereira G (2017). The association between ambient air pollution and selected adverse pregnancy outcomes in China: a systematic review. Sci Total Environ.

[12] Hastie T, Tibshirani R (1995). Generalized additive models for medical research. Stat Methods Med Res.

[13] Darrow LA, Klein M, Flanders WD, Waller LA, Correa A, Marcus M, Mulholland JA, Russell AG, Tolbert PE (2009). Ambient air pollution and preterm birth a time-series analysis. Epidemiology.

[14] Yang Y, Li R, Li W, Wang M, Cao Y, Wu Z, Xu Q (2013). The association between ambient air pollution and daily mortality in Beijing after the 2008 olympics: a time series study. PLoS One.

[15] Fell DB, Buckeridge DL, Platt RW, Kaufman JS, Basso O, Wilson K (2016). Circulating influenza virus and adverse pregnancy outcomes: a time-series study. Am J Epidemiol.

[16] Schwartz J, Dockery DW, Neas LM (1996). Is daily mortality associated specifically with fine particles?. J Air Waste Manag Assoc.

[17] Zheng Pei-wen, Wang Jian-bing, Zhang Zhen-yu, Shen Peng, Chai Peng-fei, Li Die, Jin Ming-juan, Tang Meng-Ling, Lu Huai-chu, Lin Hong-bo, Chen Kun (2017). Air pollution and hospital visits for acute upper and lower respiratory infections among children in Ningbo, China: A time-series analysis. Environmental Science and Pollution Research.

[18] Souza JB, Reisen VA, Santos JM, Franco GC (2014). Principal components and generalized linear modeling in the correlation between hospital admissions and air pollution. Rev Saude Publica.

[19] Zhao A, Chen R, Kuang X, Kan H (2014). Ambient air pollution and daily outpatient visits for cardiac arrhythmia in Shanghai, China. J Epidemiol.

[20] Tam WW, Wong TW, Ng L, Wong SY, Kung KK, Wong AH (2014). Association between air pollution and general outpatient clinic consultations for upper respiratory tract infections in Hong Kong. PLoS One.

[21] Zhang F, Wang W, Lv J, Krafft T, Xu J (2011). Time-series studies on air pollution and daily outpatient visits for allergic rhinitis in Beijing, China. Sci Total Environ.

[22] Rudez G, Janssen NA, Kilinc E, Leebeek FW, Gerlofs-Nijland ME, Spronk HM, Ten CH, Cassee FR, de Maat MP (2009). Effects of ambient air pollution on hemostasis and inflammation. Environ Health Perspect.

[23] Qian Z, Liang S, Yang S, Trevathan E, Huang Z, Yang R, Wang J, Hu K, Zhang Y, Vaughn M (2016). Ambient air pollution and preterm birth: a prospective birth cohort study in Wuhan, China. Int J Hyg Environ Health.

[24] Zeka A, Zanobetti A, Schwartz J (2006). Individual-level modifiers of the effects of particulate matter on daily mortality. Am J Epidemiol.

[25] Arroyo V, Diaz J, Ortiz C, Carmona R, Saez M, Linares C (2016). Short term effect of air pollution, noise and heat waves on preterm births in Madrid (Spain). Environ Res.

[26] Zhao N, Qiu J, Zhang Y, He X, Zhou M, Li M, Xu X, Cui H, Lv L, Lin X (2015). Ambient air pollutant PM10 and risk of preterm birth in Lanzhou, China. Environ Int.

[27] Zhao Q, Liang Z, Tao S, Zhu J, Du Y. Effects of air pollution on neonatal prematurity in Guangzhou of China: a time-series study. Environ Health. 2011;10. 10.1186/1476-069X-10-2 PMC3024279 21214958

[28] Sagiv SK, Mendola P, Loomis D, Herring AH, Neas LM, Savitz DA, Poole C (2005). A time-series analysis of air pollution and preterm birth in Pennsylvania, 1997-2001. Environ Health Perspect.

[29] Fleischer NL, Merialdi M, van Donkelaar A, Vadillo-Ortega F, Martin RV, Betran AP, Souza JP: Outdoor air pollution, preterm birth, and low birth weight: analysis of the world health organization global survey on maternal and perinatal health. Environ Health Perspect 2014, 122(4):425–430. 10.1289/ehp.1306837 PMC3984219 24508912

[30] Effect of ambient temperature and air pollutants on the risk of preterm birth, Rome 2001–2010. 10.1016/j.envint.2013.09.005 24103349

[31] Rudra CB, Williams MA, Sheppard L, Koenig JQ, Schiff MA (2011). Ambient carbon monoxide and fine particulate matter in relation to preeclampsia and preterm delivery in western Washington state. Environ Health Perspect.

[32] He JR, Liu Y, Xia XY, Ma WJ, Lin HL, Kan HD, Lu JH, Feng Q, Mo WJ, Wang P (2016). Ambient temperature and the risk of preterm birth in Guangzhou, China (2001-2011). Environ Health Perspect.

[33] Suh YJ, Ha EH, Park H, Kim YJ, Kim H, Hong YC (2008). GSTM1 polymorphism along with PM10 exposure contributes to the risk of preterm delivery. Mutat Res.

[34] Wu J, Ren C, Delfino RJ, Chung J, Wilhelm M, Ritz B (2009). Association between local traffic-generated air pollution and preeclampsia and preterm delivery in the south coast air basin of California. Environ Health Perspect.

[35] Aagaard-Tillery KM, Nuthalapaty FS, Ramsey PS, Ramin KD (2005). Preterm premature rupture of membranes: perspectives surrounding controversies in management. Am J Perinatol.

[36] Zeger SL, Thomas D, Dominici F, Samet JM, Schwartz J, Dockery D, Cohen A (2000). Exposure measurement error in time-series studies of air pollution: concepts and consequences. Environ Health Perspect.

