# Supplementary figures and images for: Association between ambient air pollutants and preterm birth in Ningbo, China: a time-series study
Source: BMC Pediatr. 2018 Sep 20;18:305. doi: 10.1186/s12887-018-1282-9 (PMC6147039; doi:10.1186/s12887-018-1282-9)

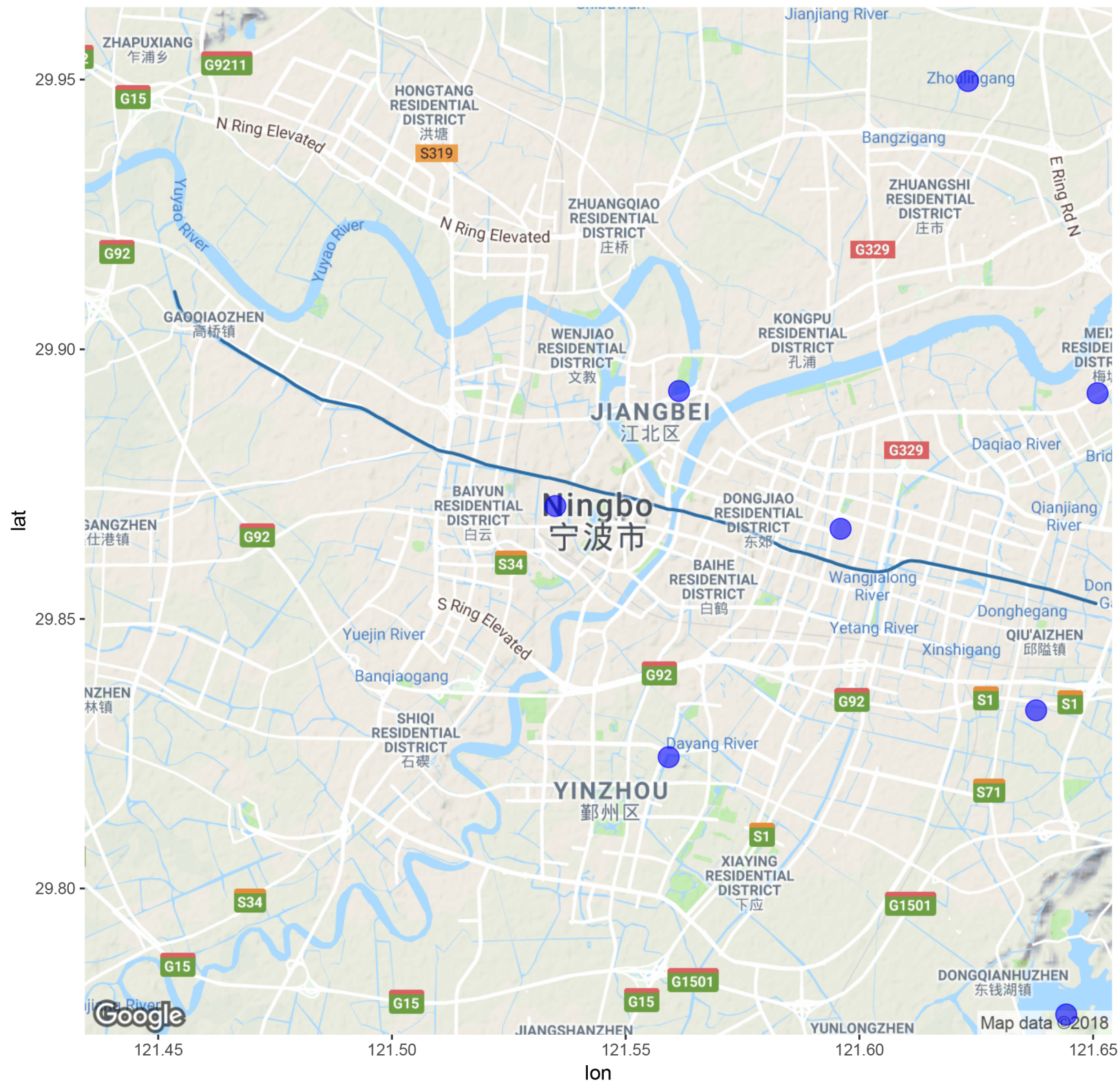

Supplemental Fig 1. Location of air quality monitor stations in Ningbo city

Supplement: Supplementary file 1 — Figure S1. Location of air quality monitor stations in Ningbo city. (PDF 13330 kb) [file 12887_2018_1282_MOESM1_ESM.pdf]
