# Supplementary material for: Association between ambient air pollutants and preterm birth in Ningbo, China: a time-series study
Source: BMC Pediatr. 2018 Sep 20;18:305. doi: 10.1186/s12887-018-1282-9 (PMC6147039; doi:10.1186/s12887-018-1282-9)
Supplement: Supplementary file 2 — Table S1. Association between cumulative air pollution concentrations and risk of preterm birth. (DOCX 18 kb) [file 12887_2018_1282_MOESM2_ESM.docx]

**Table S1** Association between cumulative air pollution concentrations and risk of preterm birth

|  | PM_2.5_ | PM_10_ | SO_2_ | NO_2_ | O_3_ | CO |
| --- | --- | --- | --- | --- | --- | --- |
| Avg 1 | 2.21 (-2.44,7.07) | 3.63 (-1.15,8.65) | 4.60 (1.78,7.49) | 5.44 (0.21,10.95) | 0.12 (-3.81,4.21) | -0.33 (-3.09,2.51) |
| Avg 2 | 2.43 (-1.36,6.36) | 3.28 (-0.57,7.28) | 2.35 (-1.06,5.88) | 6.89 (1.17,12.92) | -2.09 (-6.33,2.33) | -0.37 (-3.37,2.73) |
| Avg 3 | 4.14 (-0.11,8.56) | 4.84 (0.6,9.27) | 3.83 (0.18,7.61) | 8.85 (2.69,15.37) | -1.99 (-6.51,2.75) | 0.45 (-2.74,3.75) |
| Avg 4 | 5.60 (0.93,10.48) | 5.98 (1.37,10.8) | 4.63 (0.71,8.7) | 10.21 (3.7,17.13) | -1.51 (-6.28,3.49) | 1.51 (-1.84,4.98) |
| Avg 5 | 6.71 (1.63,12.05) | 7.01 (2.04,12.23) | 5.29 (1.13,9.62) | 10.72 (3.92,17.96) | -1.37 (-6.33,3.85) | 2.19 (-1.3,5.8) |
| Avg6 | 7.97 (2.48,13.74) | 8.56 (3.21,14.18) | 7.16 (2.69,11.82) | 11.31 (4.27,18.83) | -1.22 (-6.37,4.21) | 2.63 (-0.98,6.38) |
