# Supplementary material for: Association between ambient air pollutants and preterm birth in Ningbo, China: a time-series study
Source: BMC Pediatr. 2018 Sep 20;18:305. doi: 10.1186/s12887-018-1282-9 (PMC6147039; doi:10.1186/s12887-018-1282-9)
Supplement: Supplementary file 3 — Table S2. Excess risks (ERs) and 95% confidence intervals of preterm birth per IQR increment in air pollutant concentrations stratified by season and maternal age in Ningbo, China. (DOCX 36 kb) [file 12887_2018_1282_MOESM3_ESM.docx]

**Table S2** Excess risks (ERs) and 95% confidence intervals of preterm birth per IQR increment in air pollutant concentrations stratified by season and maternal age in Ningbo, China

| Season | Maternal age |  | PM_2.5_ | PM_10_ | SO_2_ | NO_2_ | O_3_ | CO |
| --- | --- | --- | --- | --- | --- | --- | --- | --- |
| All year | All ages | Lag 0 | 1.27(-2.05,4.7) | 1.69(-1.78,5.28) | 1.69(-1.78,5.28) | 3.86(-0.79,8.72) | -3.95(-8,0.28) | 1.5(-1.28,4.37) |
|  |  | Lag 1 | 2.36(-0.72,5.53) | 1.7(-1.86,5.39) | 1.7(-1.86,5.39) | 4.71(0.08,9.56) | -1.62(-5.51,2.43) | -0.33(-3.09,2.51) |
|  |  | Lag 2 | 2.97(-0.09,6.13) | 2.17(-1.33,5.81) | 2.17(-1.33,5.81) | 4.99(0.4,9.79) | -1.83(-5.66,2.15) | -0.28(-3.05,2.58) |
|  |  | Lag 3 | 4.84(1.77,8) | 3.56(0.07,7.17) | 3.56(0.07,7.17) | 6.49(1.86,11.34) | -0.9(-4.76,3.11) | 1.73(-1.09,4.63) |
|  |  | Lag 4 | 4.83(1.74,8.01) | 3.58(0.08,7.2) | 3.58(0.08,7.2) | 5.81(1.17,10.67) | 0.34(-3.57,4.4) | 3.36(0.5,6.3) |
|  |  | Lag 5 | 4.06(0.96,7.26) | 3.31(-0.22,6.96) | 3.31(-0.22,6.96) | 3.88(-0.76,8.73) | -0.13(-4.02,3.92) | 3.01(0.13,5.98) |
|  |  | Lag 6 | 4.05(1.04,7.14) | 3.47(0.04,7.01) | 3.47(0.04,7.01) | 4.76(0.09,9.64) | 0.12(-3.81,4.21) | 2.58(-0.33,5.57) |
|  | Maternal age between 18 and 35 | Lag 0 | 4.94(1.16,8.86) | 4.72(0.77,8.83) | 8.18(4.78,11.7) | 9.47(3.94,15.3) | -4.39(-9.05,0.5) | 3.29(0.07,6.61) |
|  |  | Lag 1 | 4.04(0.56,7.65) | 4.08(-0.03,8.37) | 5.93(2.63,9.33) | 9.96(4.44,15.77) | -4.21(-8.58,0.37) | 1.31(-1.87,4.6) |
|  |  | Lag 2 | 4.95(1.5,8.53) | 5.14(1.1,9.35) | 6.76(3.55,10.06) | 7.16(1.87,12.72) | -2.6(-6.95,1.96) | 0.17(-3,3.45) |
|  |  | Lag 3 | 4.53(1.09,8.09) | 4.88(0.88,9.03) | 6.56(3.39,9.82) | 6.66(1.42,12.17) | -2.76(-7.1,1.79) | 0.29(-2.9,3.58) |
|  |  | Lag 4 | 2.5(-0.94,6.06) | 1.97(-2,6.09) | 6.52(3.34,9.79) | 3.97(-1.22,9.43) | -0.55(-4.97,4.09) | 0.37(-2.84,3.7) |
|  |  | Lag 5 | 2.19(-1.27,5.78) | 2.12(-1.88,6.28) | 5.56(2.37,8.86) | 2.62(-2.57,8.1) | 0.02(-4.44,4.68) | 1.05(-2.2,4.4) |
|  |  | Lag 6 | 1.6(-1.77,5.09) | 1.83(-2.06,5.87) | 5.66(2.44,8.98) | 2.13(-3.04,7.57) | -1.06(-5.5,3.58) | 2.63(-0.67,6.05) |
|  | Maternal age under 18 or above 35 | Lag 0 | 8.95(-0.6,19.42) | 10.66(0.62,21.69) | 14.15(5.2,23.86) | 14.51(1.18,29.59) | -9.16(-19.7,2.76) | 7.05(0.28,14.28) |
|  |  | Lag 1 | 9.4(0.74,18.8) | 13.22(2.55,25) | 9.46(0.86,18.81) | 14.2(1.18,28.9) | -12.41(-22, 1.64) | 5.19(-1.57,12.41) |
|  |  | Lag 2 | -1.12(-9.35,7.85) | 1.9(-8.01,12.88) | 10.49(2.11,19.56) | 4.86(-7.22,18.51) | -5.76(-15.74,5.41) | 2.85(-3.86,10.04) |
|  |  | Lag 3 | -0.05(-8.29,8.94) | -0.53(-10.23,10.23) | 8.46(0.21,17.4) | 5.6(-6.67,19.47) | -4.11(-14.14,7.1) | 5.43(-1.4,12.74) |
|  |  | Lag 4 | 7.24(-1.39,16.61) | 5.75(-4.36,16.93) | 7.15(-1.07,16.05) | 8.02(-4.68,22.41) | -0.42(-10.76,11.13) | 7.82(0.85,15.28) |
|  |  | Lag 5 | 2.15(-6.3,11.37) | 0(-9.84,10.92) | 0.55(-7.58,9.4) | 7.9(-4.92,22.45) | -5.75(-15.71,5.37) | 5.84(-1.06,13.22) |
|  |  | Lag 6 | -3.38(-11.49,5.47) | -4.77(-14.23,5.73) | 3.39(-4.89,12.4) | 6.62(-6.1,21.05) | 0.45(-10.14,12.28) | 4.21(-2.71,11.62) |
| Cold period | All ages | Lag 0 | 1.01(-2.66,4.82) | 1.83(-2.09,5.91) | 0.7(-2.79,4.31) | 4.54(-1.08,10.47) | -6.47(-13.14,0.71) | 1.71(-1.87,5.42) |
|  |  | Lag 1 | 2.78(-0.69,6.37) | 3.98(-0.19,8.33) | 0.93(-2.46,4.43) | 5.97(0.33,11.93) | -3.17(-9.69,3.83) | 0.19(-3.31,3.81) |
|  |  | Lag 2 | 3.24(-0.21,6.81) | 4.51(0.39,8.78) | 1.48(-1.81,4.88) | 7.4(1.69,13.43) | -6.14(-12.44,0.61) | 0.5(-3.04,4.17) |
|  |  | Lag 3 | 4.92(1.47,8.5) | 5.11(1.04,9.35) | 4.93(1.64,8.33) | 9.25(3.45,15.38) | -6.35(-12.78,0.55) | 3.02(-0.66,6.84) |
|  |  | Lag 4 | 4.11(0.63,7.71) | 4.03(-0.02,8.24) | 3.79(0.5,7.18) | 8.22(2.43,14.33) | -5.23(-11.82,1.84) | 5.16(1.33,9.13) |
|  |  | Lag 5 | 2.74(-0.74,6.34) | 2.91(-1.16,7.14) | 2.61(-0.68,6.01) | 5.34(-0.4,11.42) | -5.14(-11.8,2.01) | 4.16(0.32,8.15) |
|  |  | Lag 6 | 3.08(-0.3,6.57) | 3.41(-0.51,7.48) | 4.06(0.73,7.49) | 7.03(1.22,13.17) | -3.94(-10.66,3.28) | 4.5(0.6,8.55) |
|  | Maternal age between 18 and 35 | Lag 0 | 5.82(1.55,10.27) | 6.66(2.11,11.42) | 7.85(3.79,12.07) | 12.18(5.49,19.29) | -4.76(-11.68,2.7) | 4.05(0.72,7.48) |
|  |  | Lag 1 | 4.2(0.23,8.33) | 6.48(1.72,11.47) | 5.74(1.8,9.83) | 12.47(5.74,19.62) | -1.63(-8.45,5.71) | 3.03(-0.28,6.45) |
|  |  | Lag 2 | 4.43(0.49,8.52) | 6.66(1.98,11.55) | 5.14(1.36,9.06) | 10.01(3.42,17.01) | -2.86(-9.61,4.39) | 0.67(-2.63,4.09) |
|  |  | Lag 3 | 4.65(0.71,8.74) | 6.74(2.12,11.58) | 7.04(3.29,10.92) | 10.32(3.69,17.37) | -3.86(-10.69,3.49) | 1.26(-2.1,4.73) |
|  |  | Lag 4 | 1.49(-2.46,5.61) | 2.42(-2.16,7.22) | 6.09(2.34,9.98) | 6.23(-0.29,13.17) | -3.49(-10.44,3.99) | 2.29(-1.16,5.87) |
|  |  | Lag 5 | -0.41(-4.36,3.7) | 0.76(-3.85,5.58) | 4.41(0.61,8.34) | 3.11(-3.34,9.99) | -4.16(-11.12,3.34) | 1.26(-2.19,4.84) |
|  |  | Lag 6 | -0.14(-4,3.87) | 1.13(-3.34,5.81) | 3.21(-0.61,7.17) | 2.49(-3.92,9.33) | -0.03(-7.25,7.75) | 4.15(0.64,7.79) |
|  | Maternal age under 18 or above 35 | Lag 0 | 6.16(-4.25,17.69) | 8.37(-2.77,20.77) | 10.17(-0.07,21.46) | 11.4(-4.37,29.78) | -16.54(-31.72,2.02) | 6.61(-1.55,15.44) |
|  |  | Lag 1 | 12.24(2.4,23.04) | 16.73(4.5,30.4) | 14.96(4.81,26.08) | 15.76(-0.32,34.43) | -15.8(-30.2,1.57) | 6.55(-1.65,15.45) |
|  |  | Lag 2 | -0.04(-9.42,10.31) | 1.94(-9.28,14.54) | 8.73(-0.89,19.28) | 5.18(-9.67,22.48) | -11.89(-26.83,6.09) | 4.79(-3.44,13.73) |
|  |  | Lag 3 | 3.72(-5.81,14.2) | 3.32(-7.89,15.9) | 10.95(1.37,21.45) | 7.4(-7.79,25.09) | -8.96(-24.85,10.28) | 8.45(-0.02,17.64) |
|  |  | Lag 4 | 10.69(0.71,21.65) | 8.41(-3.16,21.35) | 9.61(0.05,20.08) | 14.09(-2.15,33.03) | -14.83(-29.96,3.56) | 10.44(1.71,19.92) |
|  |  | Lag 5 | 1.2(-8.38,11.78) | -1.35(-12.38,11.08) | 1.48(-7.97,11.91) | 8.42(-7.34,26.85) | -21.09(-35.2,-3.9) | 6.79(-1.73,16.05) |
|  |  | Lag 6 | -5.98(-15.14,4.15) | -7.08(-17.6,4.78) | 2.66(-6.95,13.25) | 2.97(-12.15,20.68) | -13.81(-29.23,4.96) | 2.64(-5.96,12.02) |
| Warm period | All ages | Lag 0 | 0.85(-6.44,8.72) | 0.81(-6.53,8.72) | 3.94(-1.55,9.74) | 4.7(-4.31,14.57) | -1.75(-7.14,3.95) | 1.95(-2.68,6.79) |
|  |  | Lag 1 | -0.5(-7.17,6.64) | -2.59(-9.75,5.14) | 2.55(-2.76,8.14) | 4.23(-4.46,13.71) | 0.3(-4.78,5.65) | -0.59(-5.1,4.14) |
|  |  | Lag 2 | 1.11(-5.48,8.16) | -1.91(-8.95,5.68) | 4.13(-1.12,9.67) | 2.22(-6.01,11.19) | 1.61(-3.41,6.89) | -0.96(-5.45,3.75) |
|  |  | Lag 3 | 4.06(-2.55,11.12) | 1.87(-5.26,9.53) | 1.44(-3.7,6.85) | 3.12(-5.08,12.02) | 3.23(-1.81,8.53) | 0.54(-4.01,5.31) |
|  |  | Lag 4 | 6.96(0.27,14.08) | 5.01(-2.28,12.85) | 1.57(-3.59,7) | 2.77(-5.41,11.65) | 4.54(-0.51,9.85) | 1.89(-2.71,6.71) |
|  |  | Lag 5 | 7.75(1.02,14.93) | 6.88(-0.53,14.85) | 3.08(-2.11,8.55) | 2.31(-5.87,11.21) | 3.63(-1.37,8.88) | 2.13(-2.47,6.94) |
|  |  | Lag 6 | 6.88(0.19,14.03) | 5.67(-1.74,13.65) | 6.47(1.19,12.03) | 1.3(-6.86,10.17) | 3.28(-1.72,8.52) | 0.48(-4.07,5.25) |
|  | Maternal age between 18 and 35 | Lag 0 | 1.39(-6.7,10.17) | -0.93(-8.93,7.78) | 9.3(2.98,16) | 5.38(-4.58,16.38) | 1.26(-4.78,7.67) | 1.67(-3.42,7.03) |
|  |  | Lag 1 | 1.97(-5.64,10.18) | -0.14(-8.31,8.75) | 6.58(0.53,13) | 6.29(-3.39,16.94) | -0.42(-6.01,5.5) | 0.37(-4.64,5.65) |
|  |  | Lag 2 | 6.23(-1.43,14.49) | 4.37(-3.89,13.34) | 10.78(4.74,17.17) | 4.17(-4.99,14.21) | 1.28(-4.23,7.1) | 1.84(-3.27,7.22) |
|  |  | Lag 3 | 3.17(-4.23,11.15) | 2.64(-5.42,11.39) | 5.53(-0.32,11.71) | 2.7(-6.26,12.51) | 1.55(-3.91,7.32) | 0.17(-4.83,5.43) |
|  |  | Lag 4 | 5.07(-2.43,13.15) | 4.21(-3.96,13.08) | 7.53(1.64,13.76) | 2.75(-6.2,12.56) | 4.49(-1.04,10.34) | 0.4(-4.6,5.66) |
|  |  | Lag 5 | 10.5(2.71,18.89) | 10.17(1.7,19.35) | 8.58(2.65,14.86) | 5.01(-4.11,15.01) | 4.81(-0.72,10.65) | 3.54(-1.63,8.98) |
|  |  | Lag 6 | 6.32(-1.25,14.46) | 7.37(-1.01,16.46) | 11.64(5.62,18.01) | 4.29(-4.83,14.28) | 1.33(-4.08,7.05) | 3.25(-1.92,8.68) |
|  | Maternal age under 18 or above 35 | Lag 0 | 17.37(-4.7,44.56) | 18.29(-4.09,45.9) | 24.78(7.05,45.43) | 17.95(-7.85,50.98) | -3.26(-18.29,14.52) | 7.49(-4.47,20.95) |
|  |  | Lag 1 | -1.93(-18.95,18.67) | 0.65(-19.38,25.66) | -4.69(-19.58,12.96) | 7.98(-15.33,37.72) | -10.35(-23.57,5.17) | 2.28(-9.17,15.17) |
|  |  | Lag 2 | -6.05(-22.25,13.52) | 0.32(-19.5,25.02) | 16.34(-0.09,35.47) | -0.51(-21.58,26.21) | -1.31(-14.93,14.5) | -0.95(-12.04,11.54) |
|  |  | Lag 3 | -13.11(-28.12,5.03) | -15.09(-32.59,6.95) | 2.79(-12.58,20.87) | -2.94(-23.32,22.87) | -1.23(-14.53,14.15) | -0.09(-11.18,12.38) |
|  |  | Lag 4 | -3.76(-19.92,15.65) | -4.36(-23.63,19.77) | 1.76(-13.49,19.7) | -8.59(-27.93,15.95) | 8.51(-5.63,24.77) | 4.14(-7.37,17.08) |
|  |  | Lag 5 | 3.96(-13.19,24.5) | 1.81(-18.27,26.82) | -1.51(-16.55,16.25) | 1.46(-19.61,28.04) | 3.34(-10.42,19.21) | 4.54(-7,17.51) |
|  |  | Lag 6 | 2.57(-14.38,22.89) | -0.77(-20.48,23.82) | 4.86(-10.71,23.15) | 7.1(-14.91,34.82) | 9.53(-4.75,25.96) | 6.62(-5.27,20) |
