# Supplementary material for: Association between ambient air pollutants and preterm birth in Ningbo, China: a time-series study
Source: BMC Pediatr. 2018 Sep 20;18:305. doi: 10.1186/s12887-018-1282-9 (PMC6147039; doi:10.1186/s12887-018-1282-9)
Supplement: Supplementary file 4 — Table S3. Difference of estimates and 95% confidence intervals (95% CIs) of air pollutants on risk of preterm birth between subgroups. (DOCX 20 kb) [file 12887_2018_1282_MOESM4_ESM.docx]

**Table S3** Difference of estimates and 95% confidence intervals (95%CIs) of air pollutants on risk of preterm birth between subgroups

|  |  | PM2.5 | PM10 | SO2 | NO2 | O3 | CO |
| --- | --- | --- | --- | --- | --- | --- | --- |
| Season | Lag 0 | 0(-0.06,0.06) | 0.0002(-0.06,0.06) | -0.0005(-0.05,0.05) | 0(-0.07,0.07) | -0.0007(-0.06,0.06) | 0(-0.05,0.05) |
|  | Lag 1 | 0.0005(-0.06,0.06) | 0.001(-0.06,0.06) | -0.0002(-0.05,0.05) | 0.0003(-0.06,0.07) | -0.0005(-0.06,0.06) | 0.0001(-0.05,0.05) |
|  | Lag 2 | 0.0003(-0.05,0.06) | 0.001(-0.06,0.06) | -0.0004(-0.05,0.05) | 0.0007(-0.06,0.06) | -0.0012(-0.06,0.06) | 0.0002(-0.05,0.05) |
|  | Lag 3 | 0.0001(-0.05,0.05) | 0.0005(-0.06,0.06) | 0.0005(-0.05,0.05) | 0.0009(-0.06,0.06) | -0.0015(-0.06,0.06) | 0.0004(-0.05,0.05) |
|  | Lag 4 | -0.0004(-0.05,0.05) | -0.0001(-0.06,0.06) | 0.0003(-0.05,0.05) | 0.0008(-0.06,0.06) | -0.0015(-0.06,0.06) | 0.0005(-0.05,0.05) |
|  | Lag 5 | -0.0007(-0.05,0.05) | -0.0006(-0.06,0.06) | -0.0001(-0.05,0.05) | 0.0004(-0.06,0.06) | -0.0013(-0.06,0.06) | 0.0003(-0.05,0.05) |
|  | Lag 6 | -0.0006(-0.05,0.05) | -0.0003(-0.06,0.06) | -0.0003(-0.05,0.05) | 0.0008(-0.06,0.07) | -0.0011(-0.06,0.06) | 0.0006(-0.05,0.05) |
| Maternal Age | Lag 0 | -0.0006(-0.06,0.06) | -0.0008(-0.06,0.06) | -0.0008(-0.06,0.06) | -0.0007(-0.07,0.07) | 0.0008(-0.07,0.07) | -0.0005(-0.05,0.05) |
|  | Lag 1 | -0.0008(-0.06,0.06) | -0.0013(-0.07,0.06) | -0.0005(-0.06,0.06) | -0.0006(-0.07,0.07) | 0.0014(-0.07,0.07) | -0.0006(-0.05,0.05) |
|  | Lag 2 | 0.0009(-0.06,0.06) | 0.0005(-0.06,0.07) | -0.0005(-0.06,0.06) | 0.0003(-0.07,0.07) | 0.0005(-0.07,0.07) | -0.0004(-0.05,0.05) |
|  | Lag 3 | 0.0007(-0.06,0.06) | 0.0008(-0.06,0.07) | -0.0003(-0.06,0.06) | 0.0002(-0.07,0.07) | 0.0002(-0.07,0.07) | -0.0008(-0.06,0.05) |
|  | Lag 4 | -0.0007(-0.06,0.06) | -0.0006(-0.07,0.06) | -0.0001(-0.06,0.06) | -0.0006(-0.07,0.07) | 0(-0.07,0.07) | -0.0011(-0.06,0.05) |
|  | Lag 5 | 0(-0.06,0.06) | 0.0003(-0.07,0.07) | 0.0007(-0.06,0.06) | -0.0008(-0.07,0.07) | 0.0009(-0.07,0.07) | -0.0007(-0.06,0.05) |
|  | Lag 6 | 0.0008(-0.06,0.06) | 0.001(-0.06,0.07) | 0.0003(-0.06,0.06) | -0.0007(-0.07,0.07) | -0.0002(-0.07,0.07) | -0.0002(-0.06,0.05) |
